# Orbital Infarction due to Sickle Cell Disease without Orbital Pain

**DOI:** 10.1155/2016/5867850

**Published:** 2016-11-07

**Authors:** Cameron L. McBride, Kim-Binh T. Mai, Kartik S. Kumar

**Affiliations:** ^1^Ruiz Department of Ophthalmology and Visual Science, McGovern Medical School, The University of Texas Health Science Center at Houston (UTHealth), Houston, TX, USA; ^2^Robert Cizik Eye Clinic, Houston, TX, USA; ^3^Moran Pediatric Eye Clinic, An Affiliate of the Robert Cizik Eye Clinic, Houston, TX, USA

## Abstract

Sickle cell disease is a hemoglobinopathy that results in paroxysmal arteriolar occlusion and tissue infarction that can manifest in a plurality of tissues. Rarely, these infarcted crises manifest in the bony orbit. Orbital infarction usually presents with acute onset of periorbital tenderness, swelling, erythema, and pain. Soft tissue swelling can result in proptosis and attenuation of extraocular movements. Expedient diagnosis of sickle cell orbital infarction is crucial because this is a potentially sight-threatening entity. Diagnosis can be delayed since the presentation has physical and radiographic findings mimicking various infectious and traumatic processes. We describe a patient who presented with sickle cell orbital crisis without pain. This case highlights the importance of maintaining a high index of suspicion in patients with known sickle cell disease or of African descent born outside the United States in a region where screening for hemoglobinopathy is not routine, even when the presentation is not classic.

## 1. Introduction

Sickle cell disease (SCD) is a hemoglobinopathy characterized by chronic hemolytic anemia and vasoocclusive crises. It is due to a single amino acid mutation on the *β* chain of hemoglobin. The disorder is inherited in autosomal fashion, and the patients that typically experience clinical manifestations are homozygous for the sickle cell *β* globin mutation (HbSS) or compound heterozygotes with 1 sickle cell allele and an abnormality in the other allele that disallows adequate transcription of normal *β* chain. In either case, the relative amount of hemoglobin with sickle mutation (HbS) is enough to allow red blood cells to assume a sickled shape when the plasma oxygen content or pH decreases. The sickled red blood cells can occlude microcirculation and cause infarction to tissues downstream or vasoocclusive crisis [1].

Vasoocclusive crises commonly involve bone marrow of the long bones and vertebrae. Bones with less marrow space, including the bones of the orbit, are much less commonly affected [2], but vasoocclusive infarctions involving the orbit have been reported in the literature. Orbital infarction usually presents with acute onset of periorbital tenderness, swelling, erythema, and pain to the orbit. Soft tissue swelling of the orbit can result in proptosis and attenuation of extraocular movements [3–6]. Expedient diagnosis of sickle cell orbital infarction is crucial because this is a potentially sight-threatening entity [5].

We describe a case of sickle cell orbital infarction that presented without orbital pain. This has not previously been reported in the literature (determined by PubMed search on July 6, 2016, with the terms sickle cell and orbital infarction); in all previously published cases, patients complained of periorbital pain.

## 2. Case Presentation

**Figure 1:**
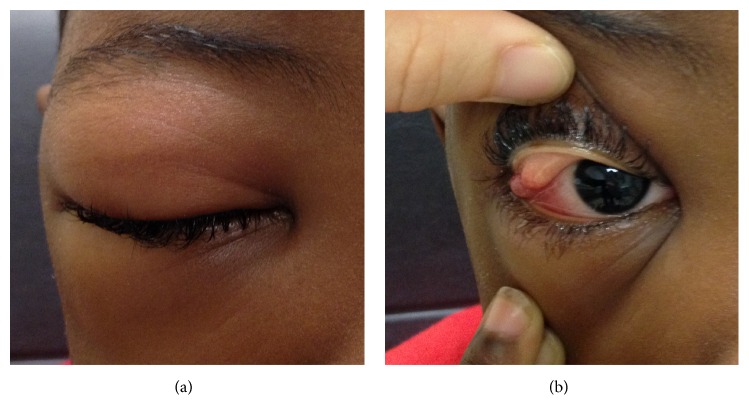
Initial presentation external photos. (a) External photo demonstrating inflammation. (b) External image with right upper eyelid everted showing fluid collection lateral to the eye.

A 5-year-old African American boy with SCD presented at Children's Memorial Hermann Hospital after 3 days of progressive swelling of his right upper and lower eyelids (Figure 1). He had no pain or mucopurulent discharge. Vision remained unchanged except for occasional double vision. He had no systemic symptoms, such as fever, rash, fatigue, or weight loss. Parents denied recent history of infection, chalazion, bug bite, or ocular or head trauma. Parents acknowledged a similar presentation in his left eye 2 years priorly, and he was diagnosed with hemorrhagic infarction of left lacrimal gland and superior ophthalmic vein thrombosis by magnetic resonance imaging (MRI). He was treated with a blood transfusion, and eye symptoms resolved after 2 weeks. He had a history of splenectomy and multiple times of hospitalization for prior vasoocclusive pain crises in the head, chest, abdomen, and hip. He was taking folate daily to maximize hematopoiesis, hydroxyurea to decrease the likelihood of sickle crises, and penicillin to prevent encapsulated bacteria.

On examination, his uncorrected visual acuity was 20/30 OD and 20/25 OS. His external exam revealed mild proptosis OD and prominent right upper and lower eyelid swelling without erythema extending to the right cheek (Figure 1). He could not open his right eye without manual manipulation. He had no pulsation, tenderness, nodularity, or induration upon palpation. His left eye was normal. His pupils measured 4 mm in the dark and 2 mm in the light OU with no relative afferent papillary defect. He had decreased motility in upgaze. The rest of the motility exam was full, and he was orthophoric. His intraocular pressures were 27 mmHg OD and 14 mmHg OS. Anterior segment exam OD revealed mild conjunctival injection and chemosis in the palpebral and temporal bulbar conjunctivae. Ophthalmoscopy revealed normal discs, macula, and vessels OU. Cup-to-disc ratio was 0.5 OU.

**Figure 2:**
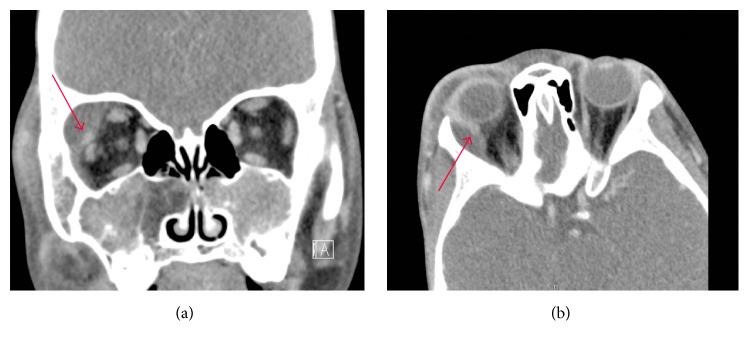
Computed tomography (CT) images. (a) Coronal CT slice exhibiting fluid collection with intraorbital mass effect (arrow). (b) Axial CT slice showing extensive soft tissue edema (arrow).

The patient was admitted given his complicated sickle cell history, high risk for infection, and concerning orbital findings. His complete blood count showed elevated white blood cells, reticulocytosis, neutrophilia, and thrombocytosis; hemoglobin and hematocrit were low. Abnormal red blood cell morphology was noted on the peripheral smear, including sickled red blood cells. Computed tomography scan of his orbits with and without contrast revealed a 27 mm × 16 mm × 12 mm rim-enhancing fluid collection in the superolateral aspect of the right orbit, displacing the lacrimal gland and orbital contents without evidence of periosteal reaction. Diffuse fat stranding was noted in the right orbit eyelids, which extended into the facial soft tissues, but no evidence of thrombosis in the vessels, sinuses, or lacrimal gland. He also had pneumatization of the maxillary sinuses with expansile bone marrow, consistent with his history of SCD (Figure 2).

**Figure 3:**
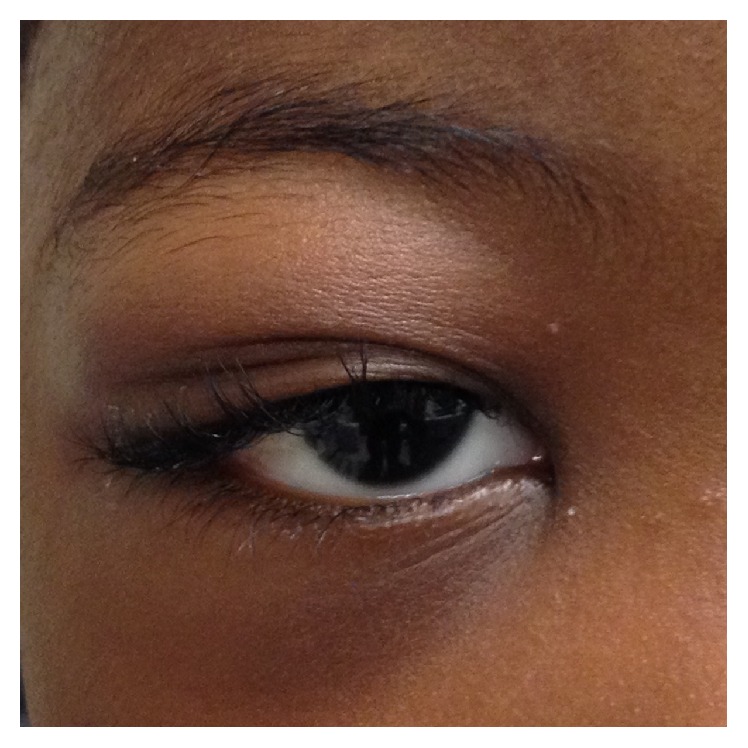
External image after resolution.

He was diagnosed with subperiosteal hemorrhagic effusion, which can occur secondary to a sphenoid wing infarction in SCD. He had an initial fever and was given intravenous clindamycin and ceftriaxone because orbital cellulitis could not be excluded based on the fluid collection with rim enhancement on computed tomography (CT). After he had remained afebrile and symptoms improved with 3 days of fluid hydration and antibiotics, he was discharged home (Figure 3).

## 3. Discussion

Orbital infarction is an uncommon manifestation of SCD that may not be initially suspected, delaying diagnosis and potentially diminishing prognosis. Further confusing the diagnosis, sickle cell orbital infarction is closely mimicked by a variety of pathologies including osteomyelitis [7] and orbital cellulitis [5]. The unique painless nature of this case and physical and CT findings rendered us unable to rule out orbital cellulitis; subsequently, empiric antibiotics were given.

Our report is the first to describe a case of painless sickle cell orbital wall infarction. One other report of orbital wall infarction without orbital pain has been described, but the patient presented with pain due to SCD in other areas of the body and was already taking pain medications when periorbital swelling began [8]. Our patient presented with no pain at all.

Patients may have proptosis due to hematomas in the orbit, which are thought to develop because of vessel wall necrosis [9]; our case was no exception. With acute swelling, pain, and erythema of the periorbital area, orbital cellulitis must be ruled out. It is not uncommon to find orbital infarction manifesting concurrently with vasoocclusive crises of anatomically distant areas [4, 9], although our patient presented with an isolated orbital crisis.

CT and MRI were used to confirm the diagnosis and identify the presence of orbital hematoma associated with orbital infarction. With MRI, infarcts will appear with rim enhancement surrounding the ischemic area, while an infectious process will have an irregular geographic enhancement where the involved bone marrow is located [10]. Surgical intervention was not considered in our case because his symptoms drastically improved before all desired imaging was completed.

The majority of cases will resolve after administration of IV fluids and medications for analgesia [11]. If the clinical picture is not distinguishable from osteomyelitis or other infectious processes in the orbit, broad-spectrum intravenous antibiotics should be administered, as was done in our patient. Many cases resolve soon after treatment with intravenous corticosteroids, which suggests that the swelling of the orbit is highly dependent on immunologic processes after the infarction. Clinicians should make a reduction in periorbital swelling a priority because of the limited space in the bony orbit and potential compromise to soft tissue structures, including the optic nerve [4]. Some authors recommend surgical drainage of hematomas [6, 12], but the majority will respond to conservative treatment and not require a procedure [3, 4].

This report highlights the importance of maintaining a high index of suspicion in patients with known SCD or of African descent born outside the United States in a region where screening for hemoglobinopathy is not routine, even when the presentation is not classic. With the techniques described above expediting diagnosis, the prognosis can be optimized and visual acuity preserved.
